# An application of the caritative caring approach – nursing students’ experiences of practising caring and uncaring encounters by simulation at a clinical training centre

**DOI:** 10.1080/17482631.2022.2100610

**Published:** 2022-07-13

**Authors:** Susanne Knutsson, Johanna Axelsson, Gunilla Lindqvist

**Affiliations:** Faculty of Health and Life Sciences, Department of Health and Caring Sciences, Linnaeus University, Växjö, Sweden

**Keywords:** Caring, encounters, nursing education, clinical training centre

## Abstract

**Purpose:**

Nurses’ lack of competence to be caring affects patients’ health and patients describe a desire for more individual and compassionate care. Nursing education tends, however, to focus less on the caring approach in nursing practice and more on developing knowledge in psychomotor skills. The aim of this study was to describe nursing students’ experiences of simulating caring and uncaring encounters founded on the caritative perspective at a Clinical Training Centre (CTC).

**Method:**

A qualitative, inductive approach using a qualitative latent content analysis. Written reflections of 49 students were analysed.

**Findings:**

By intertwining reflection with acting and observation, the students experienced that they achieved an open mind and gained an understanding of how important it was to treat the patient based on a caring approach. To act, first uncaring and thereafter caring, gave them an awakening. The students were touched and an overwhelming feeling of suddenly understanding human uniqueness and vulnerability appeared.

**Conclusions:**

To simulate caritative caring and uncaring encounters at the CTC enhanced students’ knowledge and understanding about caring and strengthened their prerequisites to acquire a caritative ontological basic view and attitude which in the long run may lead to an increased feeling of patient well-being in the encounter.

## Introduction

Patients describe that the encounter and the communication are important for quality and safety in healthcare, something also claimed by healthcare staff (Fernholm et al., 2020; Powers et al., 2017; Woods et al., 2015). However, it has been shown that nurses’ encountering and communicating skills can sometimes be questionable (Socialstyrelsen, 2022; Sundler et al., 2017) and that their lack of competence in the caring relation affects patients’ health and impacts on healthcare costs (Råberus et al., 2019). A more caritative caring approach based on compassion and the alleviation of suffering may be needed and could also be supportive when teaching nursing students how to provide a caring approach (Eriksson, 2018). Nursing education tends, however, to focus less on the caring approach in nursing practice and more on developing knowledge in psychomotor skills (Taylor et al., 2020). There is a need to find out how students can be supported in how to intertwine theory and practice to achieve both appropriation and application of a caritative ontological fundamental view and approach.

## Background

There are an increasing number of complaints made by patients regarding nurses’ lack of professional competence and compassionate care (Hogg et al., 2018; Råberus et al., 2019) and this can affect patients’ experiences (Sundler et al., 2017) and have an impact on patients’ health and well-being (Taylor et al., 2020). Nursing education today has been criticized for focusing more on developing knowledge and psychomotor skills (Cantrell et al., 2017) such as managing intravenous fluids and inserting a urinary catheter (Watts et al., 2021), or practising how to care for patients with specific diagnoses (Sterling-Fox et al., 2020) than on practising how to care for patients with a caring approach (Taylor et al., 2020). Attention is largely given to tasks or functions, rather than fundamental attitudes and approaches. To act ethically with a caring approach presupposes that one has acquired a basic ontological view and attitude (Koskinen, 2020). Eriksson (2018) is ontologically grounded in caring science and she assumes that a human being is a unity of body, soul and spirit and this holistic view includes the human being’s dignity and holiness. The inner core of her caritative caring theory comprises thoughts of love, mercy and compassion—Caritas, the Ethos. These are the basic objects of knowledge in caring science and the fundamental idea and motive to care for others is to alleviate suffering, and to promote and protect health and life (Eriksson, 2002, 2018; Eriksson & UÅ, 2007). The caring approach or the caring meeting within a caritative perspective emphasizes that the patient needs to be seen as a unique individual who is listened to, talked to, confirmed, shown respect and understanding based on a genuine, honest, committed, present, warm and loving way, a charitable treatment where the underlying action consists of a willingness to help. The goal is respect for the patient and for the patient’s dignity and holiness (Eriksson, 2018).

Theory and practical skills must be intertwined through appropriation and application and only then does caring and caring science become a natural part of ourselves in our being, our attitude, language and actions. To discover meaning and how to apply it in a particular instance are not two separate actions but one unitary process in two steps: appropriation and application in action. Appropriation is about absorbing what one has understood, incorporating the matter so that it lives within one, becomes one’s own and personal, to realize, to reach insight (Gadamer, 2013). To reach insight means a momentary conclusion where new understanding develops and transforms one’s thinking, being and acting (Sandvik et al., 2015). Application in action is about methodological procedures and approaches (Eriksson & UÅ, 2007) and where understanding becomes visible in action. If there is no appropriation and only application in action, the new understanding is reduced to a technical execution of various measures and one risks getting caught up in a repetitive act, an imitation (Sandvik et al., 2015).

Reflection is needed in order to reach understanding and to be able to intertwine theory and practice (Gadamer, 2013). Reflection starts in one’s understanding and by reflecting, one’s thoughts, feelings and experiences are considered and create order and meaning (Ekebergh, 2018). By reflecting, one understands in a new way, a new horizon takes shape and a horizon fusion has taken place, which means that the previous understanding horizon is replaced by a new understanding (Gadamer, 2013). Gadamer (2013) argues that, in the reflection, to see things in a new way, one need to be open for new things, have the skills to be open-minded and not add one´s pre-experiences or assumptions. Caring and learning have the same fundamental foundation (Eriksson, 2018) meaning, as one approach patients, one should also approach students. The use of a caring, reflective and open-minded attitude is therefore beneficial (Ekebergh, 2018; Gadamer, 2013).

Nursing education must consist of both theoretical and practical knowledge, and according to the European Union (77/453/EEC; 89/594/EEC), half of a nursing student’s study time should be dedicated to practical education. The nurse needs to independently take decisions that will allow patients to improve, maintain and recover health and to feel a sense of well-being. The nurse also needs to have an ethical and critically reflective approach and much emphasis is given to the demands of encountering, communication and teamwork (”ICN” 2021). This needs to be practised in nursing education, and the caring approach needs explicit focus in the nursing curriculum (Herron et al., 2017; Pajnkihar et al., 2020; Taylor et al., 2020). Mårtensson et al. (2021) found that students valued a caring behaviour course including simulation in a clinical training centre (CTC) with a standardized patient. Such simulation provides for students to develop a caring approach by explicit practice in caring meetings (Alden & Durham, 2017; Jarosinski & Webster, 2016). By observing other students in a simulation, students learn new skills without physically participating in the simulation. Seeing how others act in different situations and seeing new or alternative ways of caring can facilitate self-reflection and provide increased knowledge. This can also lead to students discovering that their own way of acting is not as favourable and that it can then be corrected, or lead to a confirmation that the student has the required knowledge (Abelsson et al., 2018).

Studies about learning a caring approach in nursing education can be found (Christopher et al., 2020; Knutsson et al., 2017). However, there are few studies of practising a caring approach in nursing education at a CTC (Mårtensson et al., 2021; Taylor et al., 2020). Taylor et al. (2020) and Mårtensson et al. (2021) studies are using simulations with scenarios and role-plays as tools for students learning, but other teaching strategies can probably be used as well. Some studies about how to learn the caritative caring approach in clinical education can be found (Hilli & Sandvik, 2020) but no study focusing on the caritative approach when practising at a CTC has been found. The aim of this study was therefore to describe nursing students’ experiences of simulating caring and uncaring encounters founded on the caritative perspective at a CTC.

## Method

### Design

A qualitative design with an inductive approach, using qualitative latent content analysis according to Elo and Kyngäs (2008) was chosen for the study since the aim was to gain a variety of rich and broad descriptions from individual experiences of a phenomenon to increase knowledge and provide new insights (Polit & Beck, 2017).

### Participants and setting

The participants were nursing students at a university in the southeast of Sweden. They were in their second semester and had undergone a mandatory course called “In the patient’s and the family’s world 7.5 university credits” and had participated in the course’s mandatory simulation called “Experience practice in encounter and communication” at the university’s CTC. The course had both a life-world and a caritative perspective where the simulation focused on a caritative perspective. Out of the 69 students who participated in the course, six students did not participate in the simulation due to illness and were therefore excluded since the next simulation occasion did not occur within the study’s data collection period. All 63 students that participated in the simulation were offered voluntary participation in the study whereof 49 students approved, 43 were females and six were males aged between 20–43 years (median 27 years). The students did not have any previous experience of this kind of simulation training in their education.

### Data collection

Data consisted of the students’ written, individual reflections that formed a mandatory task that had to take place the same day after the simulation and was collected via the university’s study platform during autumn 2019. The simulation was inspired by the work of Mårtensson and co-researchers (Mårtensson et al., 2021). The simulation included four different scenarios “To listen, to show compassion, to show warmth through body language and to be attendant”. Student nurses from the second semester, four assistant nursing students from a upp-secondary school nearby and three teachers (the three researchers in this study) from the university and one from the upp-secondary school participated in the simulation. The reason that assistant nursing students participated and acted as patients was to prepare the nursing students to work with other professions and to get the assistant nursing students interested in becoming a nurse. Four to five nursing students, one assistant nursing student and one teacher were grouped together. Four different groups were established with the teacher in each group concentrating on a particular scenario. This meant that only the nursing student and the assistant nursing student changed groups in order to participate in all four scenarios. In each scenario, the assistant nursing student acted as the patient, one nursing student who was the active nurse and the rest were observing the scenario and filled in an Objective Structured Clinical Examination (OSCE) document containing specific approaches needed to be taken in each scenario (Table 1). The OSCE document acted as a qualitative insurance for an objective judgment of specific skills and was helpful when later reflecting on the actions taken.

**Table 1.** An example of the OSCE protocol*.

Number of approaches	A caring encounter	X	An uncaring encounter	X	Comments
1	Listens to the patient without advancing their own assumptions / pre-understanding / views		Makes statements based on own assumptions / pre-understanding / views		
2	Is aware of how the patient seems to feel (e.g., hungry, thirsty, cold, hot, tired)		Talks despite clear signs of discomfort in the patient		
3	Uses open-ended questions appropriately		Does not use open-ended questions appropriately. Uses mostly closed questions that require only one word in response		
4	Focuses on what the patient is saying		Misses the “hooks“ the patient throws out, is not open and compliant		
5	Can be quiet		Talks all the time		
Etc … .-11.					

*The example shows caring and uncaring approaches in the scenario, To listening. In this scenario, eleven approaches were set up. The table shows five examples. The number of approaches were different in each scenario. The observing nursing students were supposed to check and comment on all the approaches during the simulation.

A description with instructions was given to all participants by the teacher i.e., “David, 82, is constantly saying that he wants to sit in his own room and eat his food there. The assistant nurse, who plays David, is determined and finds reasons why he wants to sit in his own room and not to eat in the dining room. The nurse does not listen to David at all. She is stressed and thinks he is just fussing. The nurse insists that he should eat in the dining room because then he can meet the other patients at the same time as he will get a little exercise by walking to the dining room”. Initially, the nursing student acted with an uncaring approach (for instance, the nursing student did not listen at all) towards the patient and this meeting lasted for five minutes. After that, a 15-minute long open caring period of reflection took place where the group, with the help of the OSCE document along with other nursing students’ experiences, discussed how they felt about the scenario. This meant the students were free to express their feelings and describe the exchanges in the simulation that had affected them. The teacher acted as a facilitator during the reflection and added experiences and knowledge in connection with the discussion. Thereafter, the scenario was reset, but this time the nursing student should act with a caritative caring approach (for instance, the nursing student would listen to what the patient/assistant nursing student had said) and the other nursing students observed and filled in the OSCE document once again. The scenario lasted for five minutes with a new reflection taking place which lasted 15 minutes. Each participating teacher received clear instructions about the simulation and of how the reflection should be performed and its aims. When all four simulations were completed, the nursing students were given an individual obligatory assignment (to be carried out on the same day) to write a reflection of a maximum of 250 words about the simulation. The reflection was mandatory but not used to evaluate the students. The thought was to favour a genuine reflection. The written reflection aimed to answer the questions: How did the different encounters feel? What have you learned? The students’ reflections were then sent to the university’s study platform. All 49 students sent in their reflections and they were returned on time.

Information about the study and its purpose as well as a consent form were sent by post to the nursing students two months after completing the course. The nursing students were informed that if they desired participation in the study, they should return the consent form with a signature in the pre-stamped envelope that came with the post. Information was also given verbally by teachers over Zoom at a time when the class had an introduction to another course. Two reminders were sent by email to those who did not leave a response by post and the students who wished to participate after receiving the reminder gave their consent via return email. Consent to participate meant that the researchers who were part of the study had access to the student’s reflection assignment and were allowed to use it as data in the study.

### Data analysis

A qualitative latent content analysis according to Elo and Kyngäs (2008) with an inductive approach was used to analyse the students“ experiences of caring and uncaring encounters described in the written reflections. All reflections derived from the students” study platform were compiled into a word document. According to Elo and Kyngäs (2008), the method of analysis is divided into three main phases; preparation, organization and presentation. The preparation phase started with the authors reading the material to get a deeper understanding of its content and get a picture of the whole in the collected material. Thereafter the authors identified the units to be analysed, in this case, written reflections of nursing students’ experiences of simulating caring and uncaring encounters at a clinical training centre. In the organization phase, the next step was to organize in open coding and to create categories and abstractions. Open coding means that words and sentences that answered the study’s aim, meaning units, were located. All meaning units were grouped into different preliminary categories according to similarities and differences. In the presentation phase, the preliminary categories were grouped into subcategories according to similarities and differences. By using abstraction, which involved formulating a general description of a research area, the subcategories were then sorted into generic categories according to similarities and differences. Each generic category was named according to the characteristic of its content. The generic categories were eventually abstracted into one main category capturing the essence of the students’ experiences. The content analysis resulted in seven subcategories, two generic categories and one main category.

## Ethical consideration

Both written and verbal information about the aim of the study and how the data would be collected were given to the students. The written information letter and the verbal information stated that participation was voluntary, that participants would be unidentified, and that the choice of whether or not to participate would not influence their further nursing studies, nor would it influence their grades on the present course which had already been set and which the students had already received. The students signed a consent form to participate in the study. The head of the nursing school was also informed of the study and signed a consent form. The study followed the principles of the Declaration of Helsinki (World Medical Association, 2013) and was approved by the Ethics Committee Southeast: Ref. 623–2020.

## Findings

The findings show the nursing students’ experiences of simulating caring and uncaring encounters founded on the caritative perspective at a CTC (Table 2).

**Table 2.** Findings per sub-category, generic category and main category.

Sub-category	Generic category	Main category
To act helped me understand	It gave insight and awareness	
To observe others helped me understand		
To act caring and uncaring gave an awakening		
To practice together with other professions enabled to see from different perspectives		An overwhelming feeling of suddenly understanding human uniqueness and vulnerability—the caring care was incorporated
To reflect opened the mind		
The natural self was challenged	It touched	
To dare to face		

They experienced that through acting, observation, and reflection, and they gained an understanding of how important it is to treat the patient based on a caring approach. The reflections between the two scenarios and after the last scenario gave them an open mind and helped them to gain understanding and new knowledge. To act, first uncaring and thereafter caring, produced an awakening, and acting together with assistant nurses provided useful perspectives. They achieved insight and awareness. To simulate an act, which was not compatible with the natural self, was felt to be challenging, as was facing the patient and daring to stay in the meeting. The students were touched. The nursing students described that they understood that both verbal language and non-verbal, i.e., body language, were important for a caring encounter and for the patient to be able to feel well. They felt the differences in the encounter in their body. An overwhelming feeling of suddenly understanding human uniqueness and vulnerability, the caring care was incorporated in their body.

### It gave insight and awareness

The nursing students’ experiences of acting practically, observing others, acting caring and uncaring, and practising with other professions awakened them, and intertwined with reflections, it opened their minds and increased their understanding of differences and perspectives in their patients’ vulnerability and uniqueness. This gave insight and an awareness of the importance of caring care, the willingness to do good and to help.

#### To act helped me understand

The nursing students described that by having the opportunity to act and simulate caring action and a caring approach, their understanding of the meaning of a caring encounter increased in a clear way. The students described that during the simulations they were given the opportunity to intertwine the theoretical knowledge they learned with the practical through the different role-plays and scenarios. They liked to learn about theory when it was intertwined with practice and not just sit in a classroom during a traditional lecture; it became more concrete for them. They described that they gained an increased understanding of the importance of different caring concepts as a result of the practical exercises. Some parts of the caring theory are described as easy to understand but some are described as difficult to perform in practice and therefore the students felt that it was good to be able to practice them explicitly. The nursing students describe that the simulation exercises provided experience and security and better prepared them for future clinical practice and becoming a nurse. The simulations also raised awareness of the importance of being present with their body language. According to the students, body language played an important role in the meeting with the patient, whether it was open and inviting or closed. Presenting oneself by name and title as well as a firm handshake, eye contact and having the eyes at the same level as the patient was deemed as important when creating a caring relation. It made the patient feel welcome. To ask the patient to sit down or by taking a chair yourself so that you would be at the same level as the patient further enhanced that experience. The students appreciated the simulation exercises because they made it possible for them to understand real life in a more tangible way.

*I already know how important it is to listen to the patients and keep an*

*open body position, but today it became more concrete how you actually*

*appear to the patient through your body and facial expression. I will take*

*that with me to the clinical practice.*

The nursing students felt that posture was important. They described that if they approached the patient with their arms crossed, stomped around or hung over the patient, it was perceived as an inferior encounter, it was better if they sat down next to the patient. The students highlighted that the nurse’s position of power vis-à-vis the patient can be strengthened through posture and that it is of great importance for the nurse to be aware of the patient’s vulnerability and dependent position in relation to the nurse. Tone was another factor that was mentioned by the students as important in the encounter with the patient. Through a soft but determined and middle volume tone, the students felt that they could make the patients feel calm and safe.

#### To observe others helped me understand

The nursing students described that by observing when others acted, they gained an understanding of what was caring for the patient and what was uncaring. They saw and realized how wrong it was when the nursing student did not present him/herself by name and title, which could lead to ambiguities about what questions could be asked and answered when the patient did not know who it was attending to them. The students highlighted that it was important for their understanding to first observe an uncaring encounter, to reflect on this and then observe the same scenario again but this time with a caring encounter. They could then clearly observe how the nursing student’s behaviour such as facial expressions, posture, tone of voice and attitude as well as commitment affected the patient. This highlighted differences and could help the nursing students’ understanding of the situation. They felt that they better understood how they should and should not act after they had observed.

*What I think was most important was to actually see what it can*

*look like as an observer, to see how wrong it looks when a stressed*

*nurse forgets to say hi and introduce herself. I never thought*

*about that before.*

The nursing students further describe that by observing they were reminded of how much non-verbal communication is radiated, all the time, in a meeting, and that it was a significant observation to understand how important body language is in the meeting with the patient. The students also describe that by being able to observe, they could see the scenarios from different points of view and many new thoughts were created that developed and deepened their understanding. The experiences made the students think about how they themselves wanted to meet and respond to future patients. The students described that by observing other nursing students they acquired new tools that they could benefit from in the future.

#### To act caring and uncaring gave an awakening

The nursing students experienced that by acting caring and uncaring gave them an awakening. They describe that the simulations gave rise to many new thoughts described as both inspiring and motivating. Suddenly they understood that the nurse’s attitude, for example, closeness and distance towards the patient was decisive in the encounter. The students described that they felt that they violated patient pride and deprived them of their dignity through the uncaring encounter. To practice these two dichotomies after each other made the students suddenly aware of the importance of being caring. They felt that it was a suddenly awareness to gain an understanding of how the uncaring encounter affected the patient and made them realize that they should not act in such a way towards patients in real life. They experienced that the uncaring action had a direct impact on them as an act they would never forget and would never reproduce in a real life meeting with the patient.

*Because I myself experienced an uncaring encounter, I will*

*never forget it.*

The differences in experiences between the caring and uncaring encounter helped the students to understand how important a caring approach is for one’s well-being.

#### To practice together with other professions enabled to see from different perspectives

The nursing students considered that it was satisfying to practice and reflect with teachers and assistant nursing students as everyone spoke the same language. They were engaged in the tasks and they achieved a deeper understanding of each other’s views. To be able to collaborate with assistant nursing students was experienced as a good and refreshing feeling and it was felt that there was a big difference to role-play with someone they had never met before. The collaboration was experienced as giving new views and an understanding of each other’s knowledge and how the other person thought and worked. The nursing students also experienced that the assistant nursing students also considered the simulation exercises to be instructive and that it was fun to practice together.

*… .that this was done with assistant nursing students was a big*

*plus as we are future colleagues. We are enrolled in different*

*educations but have the same goal.*

The nursing students highlighted the teachers’ presence in the exercises as important because they could communicate their experiences and knowledge to the students from their different professions. The teachers challenged the students by asking questions like, how did you think? How did it make you feel? Why did you feel that way? Could you have done something else instead? Questions of this type were experienced by the nursing students as providing them with an opportunity to see situations from different angles and gain a new understanding.

#### To reflect opened the mind

The nursing students described it as important to be able to open up their minds and their thoughts and to create an open atmosphere to be able to give and take in their reflection. They described that the reflection between the simulation exercises with caring and uncaring encounters provided them with the opportunity to ask questions and explain what they thought and have time to ponder these thoughts. Through the reflections, the students were able to share the other participants’ views, which led to new thoughts and perspectives that they had not had before.

*I thought it was good to reflect in a group and hear how others think and*

*look at things, you can then share views and thoughts with each other.*

The students said that they considered it interesting to be able to reflect with different people who had different experiences and knowledge during the simulations. The nursing students also felt that reflection was important, to have time to think about what they experienced and to reveal their own feelings. They felt that they were given time to take a step back and reflect upon and analyse their own approach to, for example, how hands were held, posture and tone of voice and how they, themselves, would like to be treated. According to the students, this self-reflection opened up for the acquisition and retention of new knowledge.

*It was interesting to play the role of the uncaring nurse and then*

*reflect on WHY it went wrong and HOW it went wrong and*

*WHAT I could have done differently.*

The nursing students describe it as useful and instructive to see and reflect on the various simulation exercises to get a clearer picture of how they treat their patients, what signals they send out and how it affects the patient. The students describe that reflection helped them to develop in the role of becoming a nurse.

### It touched

The nursing students’ experiences of first acting uncaring and then caring challenged their natural attitude and it was also about daring to stay in the encounter and meet the patient, and this touched.

#### The natural self was challenged

The nursing students described that they experienced playing the role of the nurse with a caring attitude as natural, it felt good in the whole body. They described that the benefits of the caring meeting were mutual and gave a feeling of satisfaction of having done their best and that they were happy with what they had achieved. The nursing students described that it was important for their development to play the role of a nurse with an uncaring approach to gain an understanding of how it felt and to be able to compare and feel the difference in the body and soul. Some students described emotions such as frustration and anxiety when acting in an uncaring way while others felt it as nasty and terrible. The students felt unsettled when acting as the uncaring nurse because they felt that the patient was not treated well at all. The uncaring approach was described as difficult, uncomfortable and strange and they did not feel comfortable with their actions in that encounter. It felt unnatural to not pay attention to the patient’s needs and to respond to the patient in an unworthy way by not seeing, listening, being present and not respecting the patient.

*Acting in an uncaring way did not feel good*

*at all. It felt terrible to play the role of a disrespectful, immoral and*

*unsympathetic nurse*

The students described that they experienced differences in the care they provided when a caring approach was adopted which instilled calmness and a sense of being safe for the patient which could relieve pain and anxiety. The nursing students believed that when they acted as caring nurses, feelings of satisfaction and safety emerged and that a more calm and relaxed atmosphere emerged that benefited both the patient and themselves. The students felt that they helped the patient and this feeling was experienced as natural. When the students acted in, for them, a natural way—for example, when they sat down at the same level as the patient and listened—the patient became relaxed and it was easier to have a conversation.

#### To dare to face

According to the nursing students’ experiences, it was an important component of the caring relation to dare to meet the patient in a caring way. They described that during the simulations, they experienced how the patient’s experiences of safety increased when they sat down and gave them their time, when they dared to stay in the meeting. The students also experienced how a patient would relax when they showed interest by talking and asking questions. The students explained that it was important to realize that all patients are different, that each individual is unique and that they do not want to be treated in the same way, but that it takes courage to be able to recognize each individual and meet one accordingly.

*… .I realised that good care and communication are the basis of*

*the caring relation where the patient’s suffering is*

*alleviated. A nice welcome is extremely important.*

The nursing students’ described that showing compassion is a prerequisite for good care, but that it can be confused with feeling too sorry for the patient or being sentimental. The students described, however, that it is important for the patient but also for the nurse to show compassion and that they should not be afraid to show compassion when they meet and treat patients or their relatives, it just shows that they are human.

The students’ experienced that listening was significant in the encounter. To stop talking and to have the courage to listen and be sensitive to what the patient says, and sometimes what the patient doesn’t say, was considered to be important for the safety of the patient since confidence was instilled in the nursing students when they became more involved in the patient’s world. The nursing students described that they should not be afraid of silence since it is an important part of listening. They felt that it was important to dare to be quiet since silence can allow the patient time to think and present a willingness to talk and thus strengthen the communication. By staying in the meeting and daring to listen to the patient, the nursing students described that their understanding of the patient increased and that they could understand the patient’s situation better. It was felt to be of great importance that the nursing student was aware of preconceived opinions and prejudices so that this did not become an obstacle to listening or daring to remain in listening mode.

*Being responsive and showing interest when the patient talks*

*gives you greater confidence because the person feels heard and*

*can then convey their thoughts and wishes.*

The nursing students describe daring to ask open-ended questions to the patient as something essential for the encounter. The use of open-ended questions meant that the patient thought and felt more and conveyed this to the student, who then had an increased opportunity to be able to see the whole and understand the patient. The experience was also that open questions showed the patient that the student dared to be involved, to stop and be curious about how the patient was doing and that this created safety.

## Discussion

The findings show that the nursing students’ experience of being allowed to provide an active contribution increased their understanding. Through the simulation exercises, they became aware of the importance of posture, body language and tone for the caring relation and the caring encounter in a concrete way. To practice the two dichotomies caring and uncaring encounters after each other made the students suddenly aware of the importance of being caring. They felt that it was an awakening to gain an understanding of how the uncaring encounter affected the patient. The students became aware of how theory and practice were intertwined into a whole through these exercises. They felt that this experience provided security for the upcoming clinical practice. MacLean et al. (2018) describe that being active in a safe and reality-like environment at the CTC allows students to intertwine theory and practice. It also develops their ability to communicate and be able to conduct clinical reasoning i.e., collecting information about the patient, processing it and coming to a conclusion and an understanding of the patient’s situation and giving appropriate care accordingly (Carvalho et al., 2017; Tanner, 2006).

The findings also show that observing the actions of others was experienced as a strength for their own learning. Through their observations, they became visibly aware of and gained an understanding of what a caring and uncaring encounter can mean for the patient. Abelsson et al. (2018) and Bussard and Lawrence (2019) describe that nursing students can strengthen their learning regarding care and communication by observing how fellow students act. The students can then through self-reflection acquire confirmation as to whether their own care is caring or not and be able to correct this. Stegmann et al. (2012) mean that students who observe a scenario learn as much, if not more, just by passively looking at the action and reflecting on what they are witnessing. By observing, the student can intertwine the theory and practice of caring science so that a deeper understanding can be achieved (Banneheke et al., 2017).

The findings also show that the nursing students’ experiences of daring to meet the patient, as an individual, are a prerequisite for a caring approach and the caring relation. Eriksson (2018) means that the student must show courage to dare to allow silences to occur in the caring relations so that meaningful listening is activated. It is through listening that the student can come closer to the patient and strengthen the caring relation. Being a caring nurse means being fully present in the meeting with a patient, being committed and daring to stay in the meeting (Eriksson, 2018). Bussard and Lawrence (2019) and MacLean et al. (2019) describe that it is fundamental for the caring relation to use both verbal and non-verbal communication. This can be done, for example, by seeking eye contact with the patient, touching the patient in a caring way or through conversation in a genuine manner (Jing Su et al., 2020; Söderman et al., 2018). The caring relation shapes the meaningful context of caring and stems from the ethos—caritas. To embrace love as a basic value is to make it visible in the ethical attitude and the ethical actions (Eriksson, 2018; Östman et al., 2019) and for this to happen, the carer should acquire the knowledge, but also be given the opportunity to put it into words (Näsman et al., 2008).

The findings of this study show that the students, through the simulations, suddenly realized how unique and vulnerable the patient is. Söderman et al. (2018) point out that it is of great importance to ensure each individual’s uniqueness and accept each other’s differences. Each patient is unique and must be treated individually through a holistic approach to humans (Eriksson, 2018; Jing Su et al., 2020). Eriksson (2018) believes that compassion is one of the basic concepts of caring and should not be confused with suffering yourself but suffering with someone else and this person’s situation. The students in this study described that sometimes they are afraid of the word compassion and use the word empathy instead, they are afraid of getting too close to the patient. The literature, health care programmes and health care professionals states and argues that a professional nurse should not come too close to the patient and they mean that compassion is the same as pity, associated with being weak or inferior, and something that the patient does not want to meet (Wiklund Gustin, 2017b). However, the caring literature and research describes that the patient want the nurse to come close and share the suffering, they want a compassionate nurse (Eriksson, 2018; Swanson, 1991; Valizadeh et al. (2018), Knutsson et al., 2021). Compassion is a natural quality that has been vital to humanity but has been trained away in teaching and professional practice. But, it is an important quality in the encounter with the patient (Wiklund Gustin, 2017b). Eriksson (2018) means that compassion is the source of true caring and to be able to show compassion and give a caring care one needs to be able to give, to receive and to share. Compassion requires courage, courage to take responsibility and courage to sacrifice something of oneself. Wiklund Gustin (2017a) means that compassion differs from other concepts by protecting our humanity, which relates to our responsibility as fellow human beings and our mutual dependence on others. Compassion requests and empowers the acknowledgement of the suffering of others and the action necessary to alleviate it, the other concepts do not call for action. Martinsen (2006) describes in her theory about vulnerability the importance of “seeing with the heart’s eye”. She means that compassion is an expression of mutual dependence and is a prerequisite for taking care of someone. Eriksson (2018) means that compassion is based on the caritas motive in the encounter with the suffering person, and being a nurse means meeting the patient that is suffering and that it is a natural, human reaction to be touched. The students in this study felt touched during the simulations. The natural feeling of compassion and to dare to stay in the meeting and to be present must be given more attention in nursing students’ education, both theoretically and practically and be practised in the safe environment of a CTC, in every moment and scenario, using avatars or standardized patient or using the didactic strategy used in this study; first practice an uncaring, not compassionate approach towards a fellow student, then reflect upon this, and thereafter meet the fellow student with a caring, compassionate approach and finish the simulation with a reflection. Compassion should be practiced with progress through the nursing education, to achieve an understanding that it is a natural human reaction and a part of the important caring relation.

Hustad et al. (2019) describe that nursing students through simulation exercises at a CTC can feel increased self-confidence, increase their knowledge of both technical and non-technical skills and feel more prepared for their future profession as a nurse. Kolb’s (2014) learning circle describes circular learning that involves first acting actively, which provides a concrete experience that is then reflected where new knowledge is formed through abstraction and can be tested again by actively acting. This supports the outline of the simulation exercise in this study. Eriksson (2018) means that humans learn throughout life and that learning is based on how we understand the world, how we think and what values we have, and this is dependent on how we give care (Eriksson, 2018). Therefore, it is important for the nursing student to self-reflect and be aware of own pre-assumptions to be able to see the individual patient and to give caring care (Eriksson, 2018). The findings show that the nursing students’ experiences of reflecting in connection with the simulation exercises with caring and uncaring encounters opened them up on all levels so that they became receptive to give and take, which gave room for in-depth knowledge to be implemented in their consciousness. The students felt it was necessary to be open-minded to acquire new knowledge. According to Gadamer (2013), reflection is a prerequisite for understanding and one needs to be open-minded in the reflection to achieve new understanding and knowledge. Dieckmann (2009) describes that the debriefing phase, where reflection is taking place, is the most important part of learning when simulating scenarios. A study by Lindberg et al. (2018) shows that by reflecting, the students feel that they become more aware of the value of reflecting and that it allows them to develop a reflective approach. The students also describe that being able to reflect more openly and freely is more rewarding than if the reflection is done using a strict method, as it becomes more nuanced and natural. Gadamer (2013) believes that the understanding is more limited when using a method. The students in this study thought that the teachers’ usage of open-ended questions facilitated their learning. Dahlberg and Segesten (2010) believe that the use of open-ended questions invites conversation and is important for the student’s development towards becoming a professional caregiver. In nursing education, repeated, and progressive training in reflecting could contribute to the students developing an awareness of the importance of reflection. The teachers’ role in this would be to actively ask open questions to the students, to use a clinical reasoning approach to challenge their thoughts in the various learning elements (Carvalho et al., 2017; Tanner, 2006).

The students in this study thought that the teachers’ presence, engagement and knowledge were of importance in the simulation. Since learning and caring have the same foundation, is it important for the teacher to be open-minded, reflective and possess a caritative caring approach for the students to learn. The teacher needs to bear and radiate the caring approach and in addition to that, the culture around, the environment, also needs to be caring. Caring for another human being is according to Nåden, Bergbom, Lindström and Eriksson (2018) an original idea of human love, an act of love and mercy, caritas. Caritas is an active power and a basic motive for caring and has a caring effect through its very existence in a caring culture. Claritas symbolizes clarity and is the clear light which brings about caritas. Claritas is the charismatic gleam of having assimilated the idea of caring and it acts as a guide in carrying it into effect. The teacher needs to radiate claritas in order for the students to learn a caritative caring approach and the students needs to radiate claritas in order to approach the patients in a caritative way. Nursing is a conscious act and the nursing student needs to be aware of the need for dedication, so that ethos can become visible and shown in attitudes and actions (Nåden et al., 2018).

The students in this study described that they suddenly achieved an understanding of a human’s uniqueness and vulnerability and they experienced that caring care was incorporated into their body and soul. This finding may mean that caring and caritas are carried into effect in the simulation context in this study. Gadamer (2013) means that for caring and caring science to become a natural part of ourselves in our being, our attitude, language and actions theory and practical skills must be intertwined through application, i.e., appropriation and concrete application in action (Gadamer, 2013). The application in this study contained the appropriation and the concrete application in action by the students of the caritative caring approach through the simulation scenarios at the CTC. The students’ thinking, being and acting were transformed (Sandvik et al., 2015), they incorporated the subject matter so that it began to reside within and hopefully became the students own and personal bearing (Lindström, 2014). However, we do not know, from the results in this study, that the caring manner remained within the students approach. For the caring manner to remain with the students there is a need for a surrounding caring culture ontologically grounded in the ethos caritas and for the nurse teachers’ bearing of the caritative caring science theory. There is also a need for more simulation exercises regarding caring and uncaring encounters, with the observation of others but also video recordings of themselves as pedagogical tools, and this could be implemented early in the education, preferably in the first week, and with a clear progression over the three years of education. Defenbaugh and Chikotas (2015) study shows that nursing students at an advanced level of education can also benefit from practising caring at a CTC. An appropriation and concrete application of the caritative caring approach can contribute to increased patient safety and an increased feeling of health and well-being and alleviation of suffering and hopefully then will there be a decrease in the number of complaints from patients regarding nurses’ lack of professional competence and compassionate care (Hogg et al., 2018; Råberus et al., 2019).

The students in this study observed and acted. According to Eriksson (2015, 2018) to see is to witness, and to give the knowing a speaking voice is to bear witness; “I came, I saw, and I became responsible”. We all have an ethical responsibility for uniting theory and praxis.

## Methodological considerations

The study used a qualitative design based on written reflections. Bjerkvik and Hilli (2019) mean that by using reflective writing as a didactic learning methodology, there can be a significant improvement in the student learning process through their description of the internal processes of developing self-awareness and evaluating professional experiences that may not be attainable through verbal reflection (Bjerkvik & Hilli, 2019). The questions were open-ended and the students were asked to share their experiences. Dahlberg and Segesten (2010) mean that when using open-ended questions the student can choose to write down, in their own words, what touches them to make them engaged and deepen their understanding. Methodological coherence was obtained since it was found that the research question matched the method, which matched both the data and analytical procedure. All the students in the course were asked to voluntarily participate in the study. The number is not essentially in qualitative research and the aim was seen as answered since experiences were recurring (Polit & Beck, 2017). The students may had different experiences in clinical practice, however, this is considered to not have influenced this study since the phenomenon were the students’ experiences of simulating caring and uncaring encounters at the CTC, the focus was the difference in experience of simulating caring and uncaring. The three researchers in this study participated both in the data collection and in the data analysis. However, the thought of studying this simulation occasion was decided two months after the nursing students completed the course and is not considered to have influenced the findings.

Role-play is an active learning strategy that fosters critical thinking and clinical reasoning skills and can be used in any simulation context. Students may, however, experience role-playing negatively as it is a drama technique, and therefore be reluctant to engage and/or participate. The role-play might also generate anxiety and be intimidating to students who fear appearing unintelligent or foolish. However, role-plays are application exercises and therefore require students to have some foundational knowledge and to prepare before practice and this may in itself mitigate anxiety (Nemec et al., 2021). The students in this study were given preparations and a prewritten script about the role-play. The development of this simulation assignment into a study were decided 2 months after the nurses completed the course and is therefore not seen to have influenced the results or the students´ experiences of participating in the study since the students volunteered to participate, was assured confidentiality and that they already had received credits for the course. In accordance with Polit and Beck (2017) two reminders were sent by email to those who did not leave a response. The analysis process and the results is described in sufficient detail in order for the readers to have a clear understanding of how the analysis was carried out. The results are described contents of the categories, i.e., the meanings of the categories and the content of the categories is described through subcategories and according to Elo and Kyngäs (2008) the content analysis is successful when the researcher have analysed the data and formed categories. The credibility of the findings has been reached as the findings reflect the subject of study in a reliable manner and as the categories are found to cover the data well (Elo & Kyngäs, 2008). The analysis was performed by all three researchers individually to increase comprehension, soundness of the data, interpretation and consistency. The authors’ natural attitudes regarding the phenomenon have been discussed, and one’s own values or interpretations, have been considered in order to not influence the analysis (Polit & Beck, 2017). By presenting the results in both a summary table and in a running text and confirming this with quotations, the credibility and reliability of the study has been strengthened (Elo & Kyngäs, 2008). The course had both a life-world and a caritative perspective where the simulation focused on the caritative perspective. To learn two caring perspectives can be a bias and a limit as they can be mixed by the students and as the teachers may not be ontologically grounded in the caritative perspective.

The ethical idea has been present throughout the process of this study and the ethical principles of the Declaration of Helsinki (World Medical Association, 2013) have been continuously taken into account. The students and the head of the nursing school received information about the study and signed a consent form. They were informed of the fact that they were volunteers and that they could not be identified and that they could withdraw their participation with impunity with regard to their further nursing studies and grades.

## Implications for further practice

For students to achieve knowledge and understanding in a caring approach with a caritative perspective and reach application in nursing education, at a CTC, could:

- Simulation exercises in a caritative caring approach be introduced early on in the education and be repeated with a progression throughout all semesters
- Both verbal and non-verbal caritative caring encounters be practised
- Learning didactics as acting, observing and an open reflection be useful when intertwining theory and practice
- Simulation exercises involving encounters whereby nursing students role-play being a caring and uncaring nurse be practiced
- Attention be given to the basic concepts of caring; love, mercy, compassion, and alleviation of suffering by intertwining theory in all practice situations through reflection in order to raise awareness and reach insights and be able to acquire an ontological basic view and attitude
- The need for a caritative caring culture and a bearing of caritas in the teachers be highlighted and implemented

## Conclusions

To simulate caritative caring and uncaring encounters at a CTC enhances students’ knowledge and understanding about caring. The students were touched and felt the differences into oneself. The simulations was challenging and gave an awakening. The use of reflection intertwining caritas with acting, observing and dichotomous actions like caring and uncaring deepened the students’ understanding and facilitated their awareness and insight and strengthened their prerequisites to acquire a caritative ontological basic view and attitude. To simulate caritative caring and uncaring encounters at a CTC may facilitate the students appropriation and application of a caritative caring approach. To succeed in this there is a need for a caritative caring culture and a bearing of caritas in the teachers. In the longrun this can lead to patients being encountered in a genuine caring way and that their suffering is alleviated and that the number of complaints made by patients regarding nurses’ lack of professional competence and compassionate care decreases. An interesting question for future research could be; Are the experience of caring and uncaring dependent on previous experience in clinical practice? Interviews with students about the experiences of learning caritative caring at a CTC could also be of value and also interviews with patients who have been the receiver of the caritiative caring care. To develop a tool that measures caritative caring care could be of value for both nursing education and in clinical departments.
